# Traumatic Vertebral Fracture in a Patient With Transforaminal Lumbar Interbody Fusion: A Rare Complication

**DOI:** 10.7759/cureus.19004

**Published:** 2021-10-24

**Authors:** Wan-Chi Chiang, Tsung-Hsi Tu, Jau-Ching Wu, Wen-Cheng Huang, Chao-Hung Kuo

**Affiliations:** 1 Department of Neurosurgery, Taipei Veterans General Hospital, Taipei, TWN; 2 School of Medicine, National Yang Ming Chiao Tung University, Taipei, TWN; 3 Department of Biomedical Engineering, National Yang Ming Chiao Tung University, Taipei, TWN

**Keywords:** computed tomography, vertebral facture, trauma, tlif, spondyolisthesis

## Abstract

Transforaminal lumbar interbody fusion (TLIF) offers the potential benefits of anterior and posterior column decompression and fusion. Pseudarthrosis and infection are among the most common perioperative complications. Vertebral fracture after TLIF is a rare and unusual complication. A 74-year-old female underwent L3-5 TLIF for lumbar spondylolisthesis that caused back pain and neurogenic claudication. She recovered well after surgery. However, she subsequently experienced progressive back pain and recurrent claudication after a fall. Elongated anterior-posterior length of the L5 body with progressive L5-S1 listhesis was observed in the serial radiographic follow-ups. The CT scan revealed complicated fracture lines crossing the L5 body. Further extended fixation was performed for decompression and reconstruction of the lumbosacral alignment. Although vertebral fracture after TLIF is a rare complication, a high index of suspicion is the key to early diagnosis, preferably with CT scans, for patients with traumatic accidents after TLIF surgery.

## Introduction

Lumbar spondylolisthesis, caused by degenerative disc and facet joint disease, could result in back pain, neurogenic claudication, and radiculopathy, especially in aging populations [1,2]. Surgery has been demonstrated to provide greater clinical improvement for patients with poor responses to conservative treatments, including medication, local injection, or physical therapy [2]. Transforaminal lumbar interbody fusion (TLIF) is one of the surgical options, which accesses the intervertebral space from a posterior approach. TLIF offers the potential benefits of anterior and posterior column fusion via decompressing the neural enlacements, restoring lordosis, and stabilizing the motion segment [2]. However, TLIF-related perioperative complications, including durotomy, infection, screw displacement, and implant migration, have been reported in the literature [3-5]. Durotomy and infection are reportedly the most common complications [3-5]. A higher rate of complications has been observed in patients who received multi-level interbody fusion and revision surgery [3,4,6,7].

In this report, we present a case with a rare complication; it involves a patient who presented with a trauma-related vertebral fracture after TLIF. Progressive back pain with recurrent neurogenic claudication was noted after an accident. To date, this is the first case report on a traumatic vertebral fracture on the implanted level that caused spondylolisthesis over the adjacent level and required secondary surgery for reconstruction.

## Case presentation

**Figure 1:**
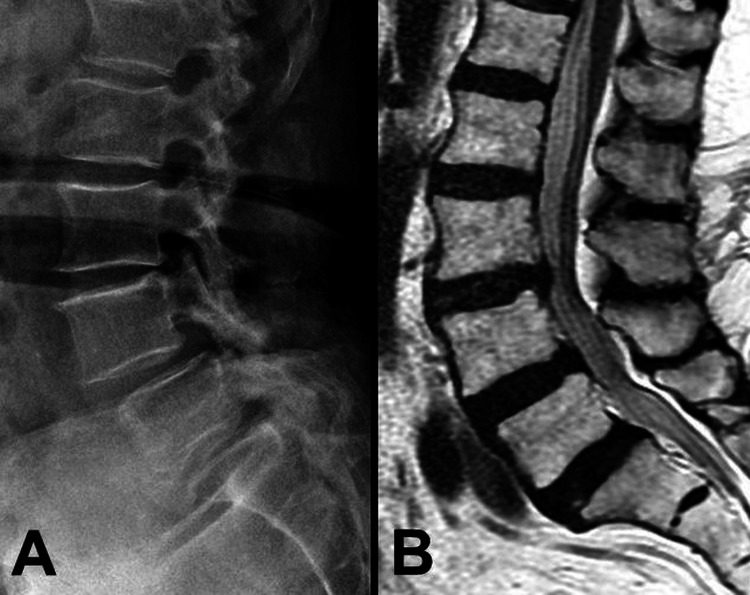
Preoperative radiograph and MRI Illustration of the preoperative radiograph with L3-4 retrolisthesis and Meyerding Grade II spondylolisthesis over L4-5 (A), and the T2-weighted MRI with spinal stenosis over L3-5 (B) MRI: magnetic resonance imaging

**Figure 2:**
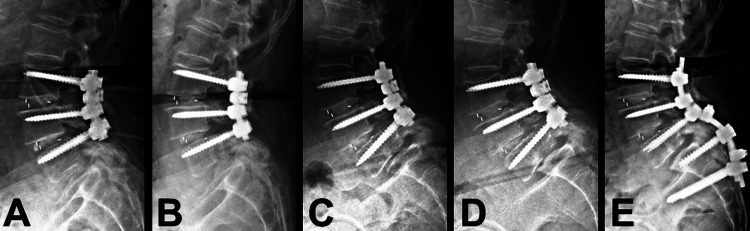
Series of radiographs during subsequent follow-ups after L3-5 TLIF The series of radiographs demonstrate the different time points of follow-up after L3-5 TLIF (A: immediate postoperative; B: two months postoperative; C: three months postoperative; and D: six months postoperative). The patient experienced a fall two months after TLIF. The radiograph arranged immediately after the episode (B) revealed no significant change, compared with the postoperative status. Elongated anterior-posterior length over L5 body with progressive listhesis was observed at postoperative three- and six-month follow-ups (C and D respectively). After the secondary surgery, L5 total laminectomy and extended fixation from L3 to S2 were performed to restore lumbosacral alignment (E) TLIF: transforaminal lumbar interbody fusion

A 74-year-old female patient had been suffering from low back pain on and off for more than two years. She presented with sciatica, numbness, and neurogenic claudication. The radiographs demonstrated Meyerding Grade II spondylolisthesis over L4-5 and retrolisthesis over L3-4 (Figure 1A). The MRI demonstrated a bulging disc with hypertrophic ligamentum flavum at the levels of L3-4-5 that had resulted in spinal stenosis (Figure 1B). Bone marrow density evaluation yielded mild osteopenia (T-score = -1.1). As she proved to be refractory to conservative treatments, the patient underwent a surgery of total laminectomy over L3-4 and L3-4-5 TLIF with the Expedium® screws and rods system (DePuy Synthes, Raynham, MA) and Capstone® cages (Medtronic, Minneapolis, MN) for interbody fusion. The immediate postoperative radiograph demonstrated satisfactory implant positions and restoration of the lumbar lordotic curvature (Figure 2A). The patient experienced significant improvement after surgery. Her back pain and numbness improved significantly. She was able to walk without using any assistive device and perform her normal daily activities.

Two months after the surgery, the patient accidentally fell on the ground. The immediate radiograph arranged after the episode, at postoperative two-month follow-up, revealed good instrumentation with persevered lumbar lordotic curvature (Figure 2B). However, progressive back pain was noted after the episode. The radiographs at postoperative three-month follow-up revealed a new onset of L5-S1 listhesis (Figure 2C). At six months after surgery, Meyerding Grade III spondylolisthesis with degenerative change over L5-S1 was observed (Figure 2D). Implant migration or screw displacement was not observed, but the anterior-posterior length of the L5 body increased during the period of further follow-ups (Figure 2). Later on, she presented with recurrent neurogenic claudication with progressive back pain.

**Figure 3:**
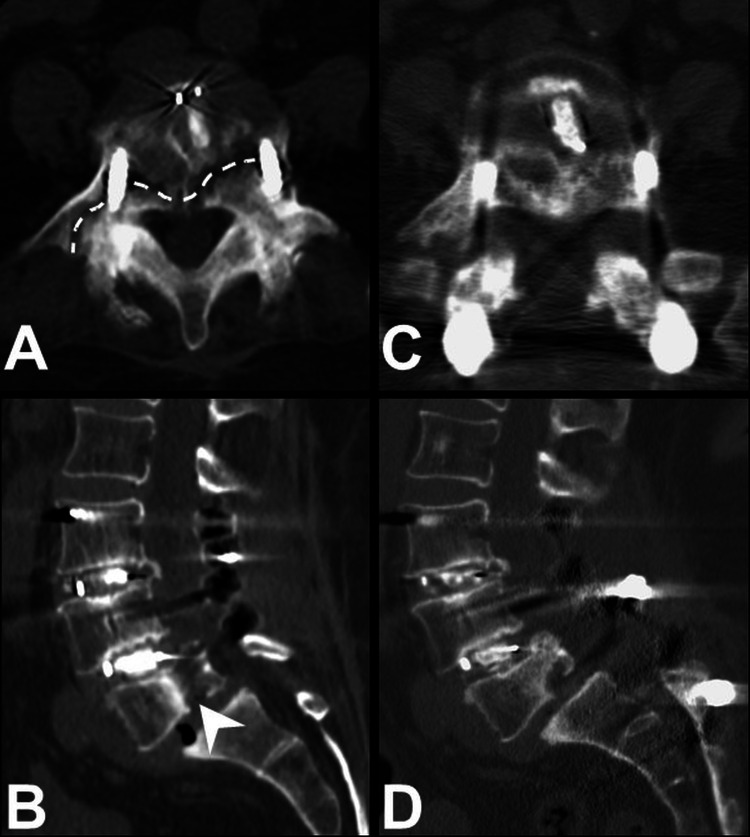
L5 fracture on CT scans Illustration of CT scans at six months after the first surgery and three months after the secondary surgery. After the patient had an accident by falling on the ground, a transverse fracture line across the right transverse to the L5 body with mild callus formation (A, dashed line), and a vertical fracture line over the posterior third of the L5 body with endplate destruction (B, arrowhead) were observed. The secondary surgery with L5 total laminectomy and extended fixation from L3 to S2 was performed. At the three-month postoperative follow-up, the CT scan revealed good bone healing over the transverse (C) and vertical fracture lines (D) after the secondary surgery CT: computed tomography

Under the suspicion of adjacent segment degeneration over L5-S1, a CT scan was arranged for further evaluation, with findings of a transverse fracture line from the right transverse process across the L5 body with mild callus formation (Figure 3A), and a vertical fracture line over the posterior part of the L5 body, extending to the lower endplate with destruction (Figure 3B). Considering the destruction of the L5 endplate with a higher risk of cage migration, a secondary surgery of L5 total laminectomy with L3-4-5-S1-2 fixation was performed. The L5-S1 listhesis that caused spinal stenosis was relieved by L5 total laminectomy, and lumbosacral curvature was reconstructed by extended fixation from L3 to S2 (Figure 2E). Fortunately, the patient's back pain and neurological symptoms improved, and she was independently ambulatory after the secondary surgery. At the three-month postoperative follow-up, the CT scan revealed good bone healing over the transverse (Figure 3C) and vertical fracture lines after the secondary surgery (Figure 3D).

## Discussion

We presented a case involving a rare complication of L5 vertebral fracture after L3-5 TLIF. The patient underwent the surgery of L3-5 TLIF due to L3-4-5 spondylolisthesis with spinal stenosis (Figure 1). After the surgery, there was a significant improvement in back pain and neurogenetic claudication. During the period of follow-ups, the patient experienced an accident by falling on the ground. Radiographs were arranged immediately after the episode and revealed no implant migration or dislocation (Figures 2A, 2B). After that, progressive back pain with recurrent neurogenic claudication was noted. A slowly developed elongated anterior-posterior length of the L5 body and of L5-S1 spondylolisthesis were observed at postoperative three- and six-month follow-ups (Figures 2C, 2D). The CT scan revealed vertical and transverse fracture lines across the L5 body (Figures 3A, 3B). Several points of view needed to be evaluated for reconstructing the lumbosacral alignment. First, considering the destructed endplate with a higher risk of cage migration [8,9], an interbody fusion between L5 and S1 was not preferred. The other consideration was if the L5 screws needed to be revised or not. The L5 body was split into several compartments by the vertical and transverse fracture lines, and carefully maintained and fixed by the current screws. Thus, surgical interventions of L5 total laminectomy to decompress L5-S1 spinal stenosis caused by listhesis, and L5 screws kept in situ with extended fixation from L3 to S2 were performed. The patient recovered well after the secondary surgery with clinical improvement.

Perioperative surgical complications of TLIF have been reported in previously published literature. In a retrospective study that included 531 patients who underwent TLIF, the most common complication was durotomy in 14.3% of patients, followed by infection in 3.8% of patients. Implant-related complications, such as screw misplacement (2.1% of patients) and cage migration (1.8% of patients) were less common [4]. A two-case series involving patients with vertebral fracture after TLIF has been reported previously. Both cases represented coronal plane vertebral body fractures in the caudal vertebral body of the TLIF construct and received secondary surgery for reconstruction [10]. The supposed risk factors included an unrecognized fracture of the endplate during cage impaction, overloading the endplates by maximizing the lordosis, or underlying mineral bone disease [10]. In our presented case, the patient had mild osteopenia. The presentations of vertebral fracture included vertical and transverse directions, and the destruction of the endplate was over the adjacent level of the cage insertion. Moreover, the patient had a history of trauma. The higher possibility of vertebral fracture may have been caused by the trauma episode.

There have been several reports that discuss the surgical intervention for spondylolisthesis over L5-S1, known as lumbosacral kyphosis. For high-grade spondylolisthesis, including Meyerding Grade III, IV, and V, the indications of reconstruction include maintaining sagittal balance, decompressing spinal stenosis, and relieving neurological deficits [11]. Diagnostic imaging, including spinopelvic parameters, should be carefully evaluated before the surgery [12]. Several surgical techniques for high-grade spondylolisthesis have been described, including (1) decompression with in situ fusion; (2) reduction with fusion; and (3) L5 vertebrectomy with fixation [13]. The techniques of fusion in situ are relatively easier to perform, with a lower risk of neurologic complications, and greater restoration of lumbosacral alignment could be offered by the technique of reduction with fusion [13,14]. However, the question as to what is the best surgical option is still controversial. To reinforce sagittal balance and lumbosacral alignment, circumferential fusion and extended fixation including S1 and S2 levels have proved to be efficient surgical strategies [11,15]. Considering the endplate condition in the presented case, interbody fusion with cage insertion may have had a higher risk of cage migration. Thus, extended in situ fixation from L3 to S2 with L5 total laminectomy for decompression was performed for the secondary surgery.

## Conclusions

TLIF offers the potential benefits of anterior and posterior column decompression and fusion for patients with lumbar spondylolisthesis. For a patient with a trauma episode after TLIF surgery, a vertebral fracture in the index level is a rare complication and should raise concern. The CT scan would be necessary for early evaluation and diagnosis. The secondary surgery for extended fixation is indicated. For symptomatic listhesis after trauma over the adjacent level, surgical intervention is also indicated for restoring lumbosacral curvature and relieving neurological deficit.
